# The prevalence of anal human papillomavirus among young HIV negative men who have sex with men

**DOI:** 10.1186/1471-2334-12-341

**Published:** 2012-12-09

**Authors:** Huachun Zou, Christopher K Fairley, Jane S Hocking, Suzanne M Garland, Andrew E Grulich, Marcus Y Chen

**Affiliations:** 1 School of Population Health, University of Melbourne, Melbourne, Australia; 2 Melbourne Sexual Health Centre, Alfred Health, Melbourne, Australia; 3 Centre for Women’s Health, Gender and Society, School of Population Health, University of Melbourne, Melbourne, Australia; 4 Department of Microbiology and Infectious Diseases, Royal Women’s Hospital and Department of Obstetrics and Gynaecology, University of Melbourne, Melbourne, Australia; 5 Kirby Institute, University of New South Wales, Sydney, Australia

**Keywords:** Human papillomavirus (HPV), Men who have sex with men, Prevalence

## Abstract

Men who have sex with men (MSM) especially those who are HIV positive are at risk for HPV-associated anal cancer. We systematically reviewed studies with data on the prevalence of vaccine preventable anal HPV among men who have sex with men aged 25 or younger and identified 6 studies. None of these studies were specifically designed to determine the prevalence of HPV in this population. Available data, albeit limited, suggest many young MSM may not already be HPV infected. Further studies using representative sampling focused on teenage MSM are required to confirm this.

## Background

Genital human papillomavirus (HPV) infection is widespread [1-3] and usually asymptomatic [4]. HPV types 6 and 11 are the types most commonly associated with anogenital warts [5], while types 16 and 18 are associated with HPV related malignancies including anal and cervical cancer [1-3,6]. Studies demonstrate that the great majority of adult MSM are infected with multiple HPV types [7-9], with a substantial proportion infected with HPV 16 or 18, which together cause about 80% of anal cancers [10-12]. MSM especially those who are HIV positive are at increased risk for anal cancer [13].

The prophylactic quadrivalent HPV vaccine is effective in preventing infection with HPV types 6, 11, 16 and 18 and in reducing the incidence of anogenital warts and anal intraepithelial neoplasia in males [14,15]. However, to date no countries have introduced universal, free vaccination of boys. The extent to which young MSM are already infected with HPV at the age at which targeted vaccination is available is not well documented. We therefore undertook a review of studies examining the prevalence of HPV among MSM aged 25 and younger.

## Methods

We systematically reviewed studies by searching MEDLINE and abstracts from the European Research Organisation on Genital Infection and Neoplasia (EUROGIN) Conferences and the International Papillomavirus (IPV) Conferences. Key words used in the search included: “men who have sex with men” or “MSM” or “homosexual” or “bisexual” or “gay” and “human papillomavirus” or “HPV”.

The following data were extracted: author, year of publication, study setting, number of MSM, number of MSM aged 25 or younger, sample collection method, HPV DNA testing method, and HPV type tested. HPV 6/11/16/18 prevalence were extracted, and where possible, HPV 6/11/16/18 prevalence stratified by age group (≤20 years old; 21–25 years old; ≤25 years old).

## Results

**Figure 1:**
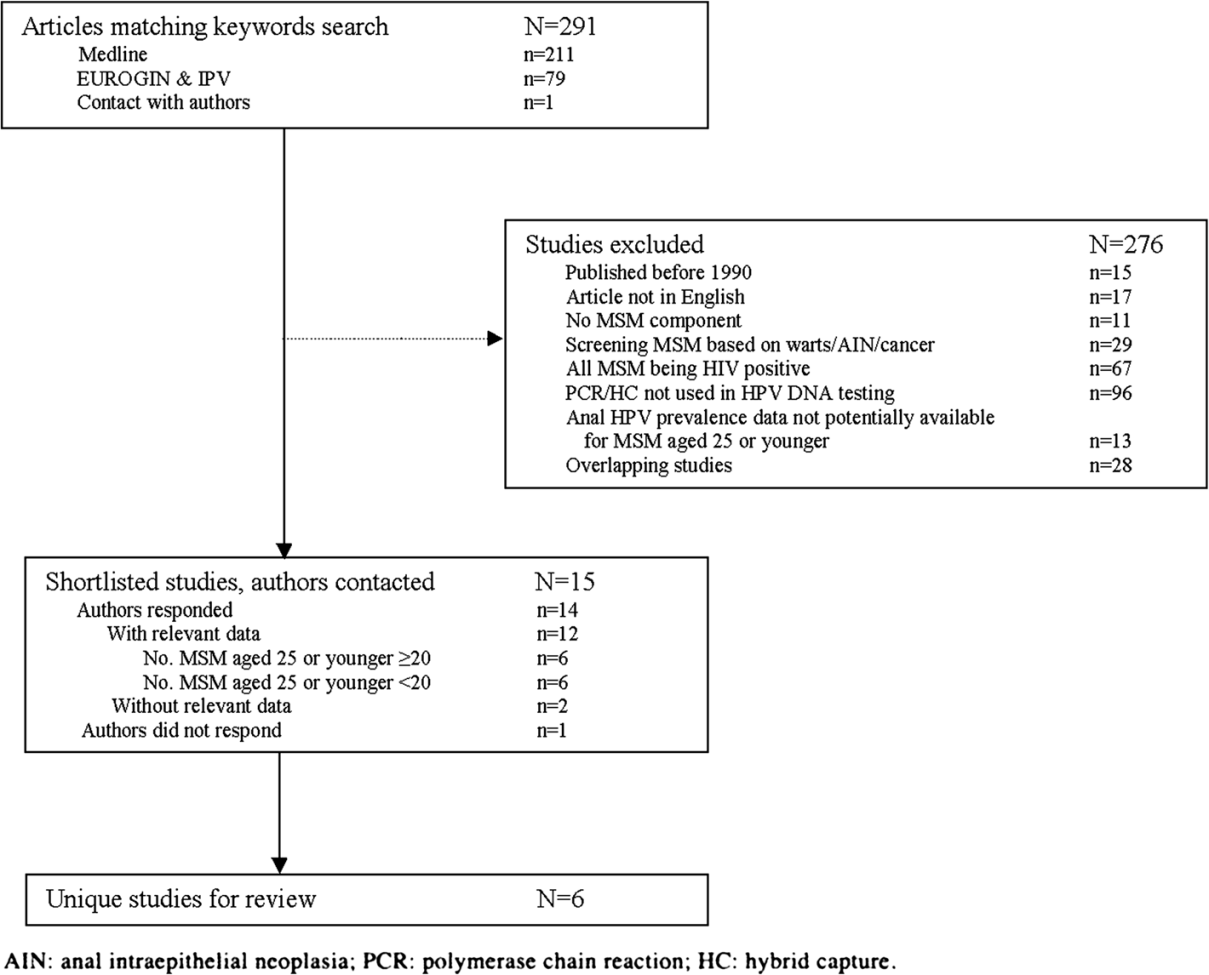
**Selection procedure for studies of anal HPV prevalence among HIV-negative MSM25 years.** AIN:anal intraepithelial neoplasia; PCR: polymerase chain reaction; hybrid capture.

The outcome of the review is summarized in Figure 1. The study populations, selection criteria, testing methods for HPV and HPV types covered in the 6 studies included are presented in Table 1. Importantly, none of the studies specifically sought to determine the prevalence of HPV infection among a representative sample of young MSM [7,16-19].

**Table 1:** Overview of studies with data on anal HPV detection among men who have sex with men aged ≤ 25 years

**Author, year**	**Study setting**	**Selection criteria**	**No. MSM**	**No. MSM <=25**^a^	**Sample collection method**	**HPV DNA testing method**	**HPV types tested**
(i) Kiviat, 1993 [16]; (ii) Critchlow, 1998 [17]	(i) Homosexual men attending an STD clinic in Seattle (ii) Homosexual men attending a community-based clinic for HIV screening in Seattle	(i) Age 16–50; being homosexual or bisexual. (ii) Age ≥18; having sex with other men	854	58^b^	Clinician collected	PCR; primers MY09 and MY11	PCR: 6,11,16,18,31 33, 35,39 and 45
Moscicki, 2003 [18]	Adolescents in primary care centres in 13 US cities who were at high risk of HIV infection	Age 13–18; having high-risk behaviours and/or injecting drug use	83	25^c^	Clinician collected	PCR, primers MY09, MY11 and HMB01	16,18,31,33,39,42, 44,45,51,52, 56 and 58
Nyitray, 2011[7]	MSM from the general population, universities, STD clinics and organized health care systems in Sao Paulo, Cuernavaca, Tampa.	Age 18–70; having no prior penile or anal cancer or genital warts and no current HIV/STD diagnosis	176	46	Clinician collected	PCR;the QIAamp Media MDx kit (Qiagen); primers PGMY09/11	6,11,16,18,26,31,33, 35,39,40,42,45, 51–56,58,59,61,62,64, 66–73,81-84, IS39 and CP6108
Gilbert, 2011 [20]	MSM recruited from bars, festivals, associations, community events, bathhouses, and businesses in Vancouver	Age ≥19; identifying as a man who has ever had sex with men	178	30	Self collected	PCR; PCR and Linear Array kit (Roche); primers PGMY.	36 types including 6,11,16,18
Goldstone, 2011[19]	MSM in a randomized, placebo-controlled, double-blinded trial of HPV vaccination in 14 countries	Age 16–26; having ≤ 5 lifetime male and/or female sexual partners; having had penetrative intercourse, including oral sex	602	539	Clinician collected	PCR; QIAamp DNA kit (Qiagen); primers based on published L1, E6 and E7 sequences.	6,11,16,18, 31,33,35,39, 45,51,52, 56, 58 and 59

Note:

^a^ Number of HIV negative MSM ≤ 25 years old for whom data on anal HPV DNA was provided by the authors.

^b^ Combined data from two separate studies (i and ii) provided by the author.

^c^ 58 men who were HIV positive were excluded.

The 6 studies included provided data on anal HPV for a total of 698 MSM aged ≤ 25. Among men aged ≤ 20, the prevalence of anal HPV 6, 11, 16 and 18 ranged from 0 to 17%, 0 to 17%, 0 to 14% and 0 to 20%, respectively. Among men aged 21 to 25, the prevalence of anal HPV 6, 11, 16 and 18 ranged from 8 to 19%, 0 to 8%, 0 to 18% and 3 to 8%, respectively.

## Discussion

Through this review, we found that there have been no published studies that have been designed to specifically determine the prevalence of HPV among young MSM using a representative sample of young MSM. Of the six studies identified, all had potential biases because of the way the studies were designed. These could have led to either an overestimate or an underestimate of the true HPV prevalence among young MSM. Several studies recruited men from clinics including STD clinics [7,17,18] while others had selection criteria that may have led to higher risk or lower risk men being included [15,19]. Several studies recruited MSM across a wider age range leaving only a much smaller subset of younger MSM with data on HPV prevalence. Most data derive from a study that was designed to determine HPV vaccine efficacy in MSM rather than HPV prevalence [15]. A further limitation is that all studies only reported HPV detection at a single time point rather than defining infection by the presence of the same HPV type present at two separate time points.

Despite these important limitations, the available data suggest HPV infection may still be uncommon among teenage same sex attracted males, although the data are currently insufficient to draw this conclusion with confidence. A community-based, representative sample is needed to determine the prevalence of HPV infection, defined rigorously, among mainly teenage same sex attracted males.

Given that HPV 16 and 18 are responsible for around 80% of anal cancers [12] and HPV 6 and 11 account for most genital warts [21] such a study would help to ascertain whether targeted HPV vaccination of young MSM is likely to be effective in preventing anal cancer in this group of at-risk males. Ideally, HPV vaccination should occur prior to the onset of sexual activity – ahead of potential HPV acquisition. To date, however, no country has implemented universal HPV vaccination of school aged boys, although the Australian Government has recently announced it is planning to roll out the HPV vaccination program to 12 and 13 years old schoolboys from 2013. Targeted vaccination of MSM to prevent anal cancer would likely only be effective if, in addition to low existing HPV rates, young same sex attracted males are willing to disclose their sexuality to health care providers in order to obtain the HPV vaccine. Further studies to determine the acceptability and feasibility of targeted vaccination of young same sex attracted males are required.

## Abbreviations

HPV: Human papillomavirus; MSM: Men who have sex with men; EUROGIN: European Research Organisation on Genital Infection and Neoplasia; IPV: The International Papillomavirus Society; STD: Sexually transmitted diseases.

## Competing interests

CF has received honoraria from CSL Biotherapies and Merck and research funding from CSL Biotherapies. CF owns shares in CSL Biotherapies the manufacturer for Gardasil. JH has received an honorarium from CSL Biotherapies and is an investigator on an Australian Research Council funded project (LP0883831) that includes CSL Biotherapies as a research partner. AG has received honoraria and untied research funding from CSL biotherapies, and has received honoraria from Merck. SG has received advisory board fees and grant support from CSL and GlaxoSmithKline, and lecture fees from Merck, GSK and Sanofi Pasteur; in addition, she has received funding through her institution to conduct HPV vaccine studies for MSD and GSK. SG is a member of the Merck Global Advisory Board as well as the Merck Scientific Advisory Committee for HPV. None of this relates to this specific work. MC and HZ have no conflicts of interest.

## Authors’ contributions

HZ and MC carried out the literature review and drafted the article. JH, SG, KF and AG participated in the coordination and editing of the article. All authors read and approved the final manuscript.

## Pre-publication history

The pre-publication history for this paper can be accessed here:

http://www.biomedcentral.com/1471-2334/12/341/prepub
